# Comparison of Risk Assessment Strategies Recommended by the 2019 and 2024 European Society of Cardiology Guidelines in Patients With Stable Chest Pain: A Coronary Computed Tomography Angiography Study

**DOI:** 10.31083/RCM50177

**Published:** 2026-09-18

**Authors:** Hantao Jiang, Chang Liu, Chao Zhang, Chengjian Wang, Minghui Wang, Jia Zhao, Jia Zhou

**Affiliations:** ^1^Department of Cardiology, Tianjin University Chest Hospital, 300222 Tianjin, China; ^2^Department of Intensive Care Unit, Baodi Hospital Affiliated to Tianjin Medical University, 301800 Tianjin, China; ^3^Department of Emergency, Tianjin Jinnan Hospital, Tianjin University, 300350 Tianjin, China

**Keywords:** risk assessment strategies, European Society of Cardiology guidelines, stable chest pain, coronary computed tomography angiography, chronic coronary syndrome

## Abstract

**Background:**

The European Society of Cardiology issued guidelines in 2019 and 2024 to help clinicians determine whether patients with stable chest pain (SCP) require advanced cardiovascular imaging testing (CIT). This study aimed to compare the safety and effectiveness of these evaluation strategies.

**Methods:**

A total of 17,612 symptomatic patients referred for coronary computed tomography angiography (CCTA) were included and followed over time. Each patient was classified into a deferral or referral group according to the 2019 and 2024 strategies, respectively. Obstructive coronary artery disease (CAD) was primarily defined as a lesion with ≥50% diameter stenosis on CCTA. Associations between major adverse cardiovascular events (MACEs) and the deferral and referral groups were evaluated using hazard ratios (HRs). Net reclassification improvement (NRI) and the number needed to test (NNT) to prevent unnecessary CIT were used to compare the two strategies.

**Results:**

Under the 2019 and 2024 strategies, 40% and 46% of patients, respectively, were categorized into deferral groups. These groups demonstrated a low prevalence of obstructive CAD (9.8% and 9.4%) and MACE (3.9% and 3.9%), suggesting that only limited intensive clinical follow-up was needed. Compared with the 2019 strategy, the 2024 strategy revealed a stronger association with MACEs (HR: 3.64 vs. 3.28) and a positive NRI (6.11%; *p* < 0.001), resulting in an NNT of 17.

**Conclusions:**

The 2024 strategy maintained a safety profile comparable to that of the 2019 strategy while potentially reducing unnecessary CIT in patients presenting with SCP suggestive of chronic coronary syndrome (CCS).

## 1. Introduction

Stable chest pain (SCP) is highly prevalent, with a lifetime risk approaching 40%, driving millions of evaluations for chronic coronary syndrome (CCS) annually [1,2]. The use of cardiovascular imaging testing (CIT) to confirm or exclude obstructive coronary artery disease (CAD) in the assessment of SCP has risen sharply over the last decade [3,4,5]. Moreover, newer diagnostic perspectives, such as epicardial adipose tissue, provide more information beyond stenosis assessment [6]. Consequently, establishing criteria for which patients with CCS necessitate further CIT versus those who can be safely deferred remains a cornerstone of guideline-directed management [1,2].

To better identify patients for CIT, the 2019 European Society of Cardiology (ESC) CCS guidelines incorporated the concept of pretest probability (PTP) of obstructive CAD [7], specifically endorsing the updated ESC-PTP model [8]. In this framework, CIT may be deferred when the ESC-PTP is <5% and is indicated when the ESC-PTP is >15%. To better assess patients in the borderline risk range (5–15%), the guidelines introduced the metric of clinical likelihood (CL) [7]. Subsequently, using data from 41,177 symptomatic patients undergoing coronary computed tomography angiography (CCTA), Winther et al. [9] proposed the risk factor-weighted CL (RF-CL) model to estimate CL. Since then, the RF-CL model has been externally validated and compared to ESC-PTP in different CCTA-based cohorts of patients with SCP [10,11,12,13], as well as in our earlier work using the CCTA Improves Clinical Management of SCP (CICM-SCP) registry [14,15]. To ensure safe and effective risk assessment, the 2024 ESC guidelines notably replaced the ESC-PTP model with the RF-CL model [2]. Under these guidelines, further CIT—such as CCTA—is recommended exclusively for patients with a CL >5% [2].

Currently, there is a lack of research comparing cardiovascular risk assessment strategies outlined in the 2019 and 2024 ESC guidelines, particularly in the Chinese population. Using data from the CICM-SCP registry, this study systematically compared the safety and effectiveness of the two risk-stratification strategies for patients with SCP suggestive of CCS who are unlikely to benefit from CIT. Specifically, it compared the 2019 strategy—which applied the RF-CL model solely to patients with borderline ESC-PTP—against the 2024 strategy, which applied the model universally to all patients.

## 2. Methods

### 2.1 Study Population

This study was a post hoc analysis of the CICM-SCP registry, which is an ongoing, prospective, multicenter cohort of patients who were referred for CCTA as first-line CIT for the assessment of SCP suggestive of CCS (ClinicalTrials.gov Identifier: NCT04691037). Details about this cohort have been previously described [14,15,16]. A total of 17,612 patients undergoing CCTA between August 2016 and August 2018 were analyzed after sequential exclusion of individuals with acute coronary syndrome, pre-existing CAD, structural heart disease, or heart failure. Missing baseline records, and patients over 90 years old or those with non-sinus rhythm were also excluded (Fig. 1). All patients were followed up until August 2023. This observational study complied with the Declaration of Helsinki and was approved by the ethics committees of local institutions.

**Fig. 1.**
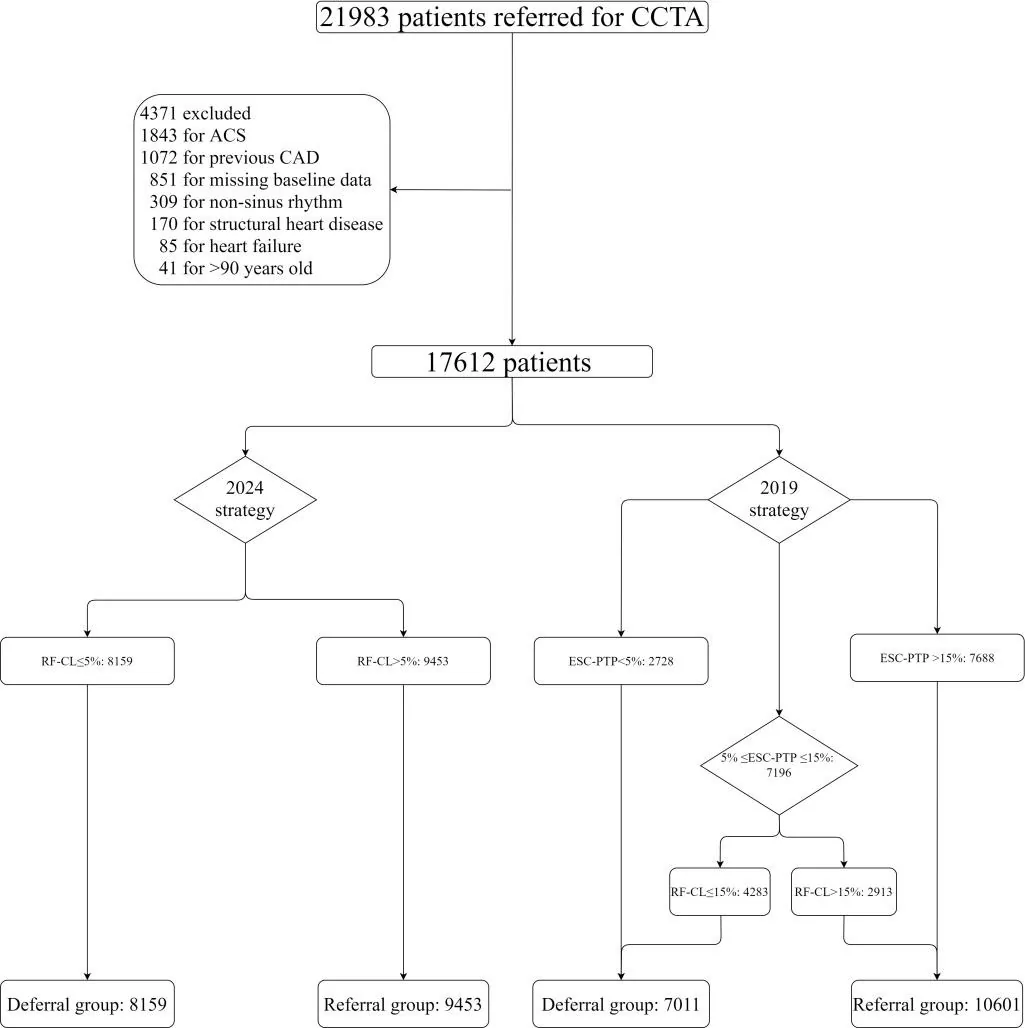
**Flow diagram**. CCTA, coronary computed tomography angiography; ACS, acute coronary syndrome; CAD, coronary artery disease; ESC, European Society of Cardiology; PTP, pretest probability; RF-CL, risk factor-weighted clinical likelihood; 2019 strategy, risk assessment strategy recommended by 2019 European Society of Cardiology guidelines; 2024 strategy, risk assessment strategy recommended by 2024 European Society of Cardiology guidelines.

### 2.2 Baseline Characteristics

Baseline demographic and clinical characteristics—including age, sex, diabetes, hypertension, hyperlipidemia, smoking, family history of CAD, and symptom type (typical angina, atypical angina, or nonanginal chest pain)—were collected and defined in accordance with previous publications [14,15,16]. Smoking was defined as current smoking or smoking within the previous 6 months. A family history of CAD was considered positive if a first-degree male relative had been diagnosed before age 55, or a first-degree female relative before age 65. Typical angina is defined by the presence of all three classic features [7]: (1) substernal discomfort of characteristic quality, (2) triggers like physical exertion or emotion, and (3) relief with rest or nitroglycerin within 10 min. Atypical angina requires two of these features, while nonanginal chest pain meets only one or none.

### 2.3 Risk Assessment Strategies

For each patient, ESC-PTP was calculated based on age, sex, and symptoms [7], while RF-CL was determined using age, sex, symptoms, hypertension, dyslipidemia, diabetes, smoking, and a family history of CAD [2]. Based on their risk assessment, patients were classified into a deferral group (no further CIT) or a referral group (further CIT). In accordance with current guidelines [1,2,7] and emerging evidence [10,11,12,13,14,15], details of the two risk assessment strategies are presented below and in Fig. 1.

According to the 2019 strategy, patients with ESC-PTP <5% were classified as the deferral group, and patients with ESC-PTP >15% were classified as the referral group. For patients with a borderline ESC-PTP score (5–15%), an RF-CL >15% indicates referral for evaluation, while an RF-CL ≤15% qualifies for deferral. Among patients with borderline ESC-PTP, previous studies have shown that RF-CL ≤15% indicates a very low risk of obstructive CAD and future clinical events [10,11,12,13,14,15]. Therefore, we selected a 15% RF-CL cutoff to optimally balance safety and effectiveness when deferring CIT. According to the 2024 strategy, patients with RF-CL ≤5% were assigned to the deferral group; all other patients were assigned to the referral group.

### 2.4 Coronary Computed Tomography Angiography (CCTA)

The CCTA scans and interpretations were performed following established guidelines [17] and local protocols as previously described [14,15,16]. CCTA images were acquired using a CT scanner with ≥64 detector rows. All scans were independently analyzed by three experienced observers, two radiologists, and one cardiologist, who were blinded to the data. All interobserver disagreements were resolved by consensus. According to the Coronary Artery Disease–Reporting and Data System [17], all segments ≥2 mm in diameter were identified and analyzed. Luminal stenosis was categorized as 0%, 1–49%, 50–69%, and >70%. Obstructive CAD was primarily defined by the presence of ≥50% diameter stenosis in at least one lesion, whereas nonobstructive CAD was defined as 1–49% diameter stenosis in the absence of any ≥50% lesion. Similarly, obstructive CAD was defined as the presence of at least one lesion with ≥70% diameter stenosis, whereas nonobstructive CAD was defined as having at least one lesion with 1–69% diameter stenosis with no lesion reaching ≥70%. Patients were classified as having no CAD if they showed 0% diameter stenosis in all lesions. An unnecessary CIT was defined as a CCTA revealing <50% diameter stenosis, as both the ESC-PTP [8] and RF-CL [9] models were mainly developed using a ≥50% stenosis threshold to assess risk in patients with SCP suggestive of CCS. Conversely, clinical interventions like revascularization are typically guided by a threshold of ≥50% stenosis. While a >0% cutoff is highly sensitive, it generally indicates subclinical atherosclerosis seen in primary prevention.

### 2.5 Follow-up and Endpoints

As previously described [14,15,16], patients were tracked through physician visits, mail, or phone, unless they were deceased or lost to follow-up. An independent clinical event committee, blinded to all other data, evaluated all endpoints. The primary endpoint was a major adverse cardiovascular event (MACE), defined as the combination of all-cause mortality and nonfatal myocardial infarction (MI). All-cause death was chosen over cardiac death to avoid difficult cause-of-death adjudications—particularly since mortality rates were low—although specific causes of death in the registry were still investigated. MI was defined according to the Fourth Universal Definition of Myocardial Infarction [18]. Cardiovascular death included death resulting from MI, sudden cardiac death, heart failure, stroke, cardiovascular procedures, cardiovascular hemorrhage, and other cardiovascular causes [19]. Unwitnessed deaths or those lacking a clear cause were classified as cardiovascular mortality.

For each patient, we captured increases in secondary prevention medications for CAD (antiplatelet, anti-ischemic, lipid-lowering, and renin-angiotensin-aldosterone system blockers), invasive coronary angiography (ICA), and revascularization procedures performed within 60 days of the index CCTA [14,16]. This 60-day cutoff was chosen based on local practices, the natural history of CCS [20,21,22], and our previous studies [14,16] to identify downstream diagnostic and therapeutic decisions likely triggered by the index CCTA. Unplanned revascularization, which is usually driven by unanticipated disease progression or inadequate symptom control, was not attributed to the index CCTA. However, due to the nature of the data sources, we were unable to reliably distinguish planned from unplanned revascularization. Therefore, revascularization occurring >60 days after the index CCTA was defined as unplanned to minimize verification bias. The secondary endpoints included all-cause death, cardiovascular death, nonfatal MI, downstream diagnostic, and therapeutic decisions attributable to index CCTA (e.g., medication increase, ICA, and revascularization), and late revascularization.

### 2.6 Statistical Analyses

All statistical analyses were performed by R (version 4.0.3; R Foundation for Statistical Computing, Vienna, Austria) or MedCalc (version 15.2.2; MedCalc Software, Mariakerke, Belgium). Two-tailed *p* < 0.05 was considered statistically significant. Kaplan-Meier curves were generated for cumulative event-free estimated survival from the primary endpoint and were compared using the log-rank test. Cox proportional hazard regression models were used to calculate hazard ratios (HRs) and 95% confidence intervals (CIs), which allowed assessments of how different strategy-based groups were associated with the time to first endpoint. Using the methods outlined by Altman et al. [23], the HRs and associated *p* values for the two strategies were compared. The powerSurvEpi R package was used to estimate the sample size for the observational cohort study investigating the association between groups and time to MACE. Based on an expected HR of 2 (referral vs. deferral group) and a referral group proportion of 40%, we set the 5-year event-free survival in the deferral group at 4%. To achieve 80% power with a two-sided significance level of α = 0.05, the required number of events was estimated to be 68.

The Student’s *t*-test was used to compare normally distributed continuous data, and the Mann-Whitney U test was used to compare nonnormally distributed continuous data. Differences in the percentages were compared using the χ2 test or Fisher’s exact test as appropriate. To further validate the effectiveness of our risk assessment strategy, we evaluated the diagnostic yield, which was defined as the proportion of patients who had positive findings on their CCTA. A reclassification table was used to calculate the net reclassification improvement (NRI), measuring how effectively one strategy reclassified patients into risk groups compared to another [24]. According to the reclassification table, the number needed to test (NNT) was defined as the number of patients needed to undergo testing in order to reclassify one patient from one group to another [12]. Due to the absence of controls in the present study, the NNT was calculated as the inverse function of the difference between the correct and incorrect reclassification rates. Thus, the NNT measures a strategy’s absolute impact by factoring in baseline risk. This prevents the exaggerated effects often seen when only relative measures are reported in real-world settings.

## 3. Results

### 3.1 Study Population and Baseline Characteristics

As shown in Fig. 1, under the 2024 strategy, 46% (8159/17,612) of patients included in the final analysis were assigned to the deferral group. Among the 7196 patients with an ESC-PTP between 5% and 15%, 4283 had an RF-CL ≤15%. Together with patients with an ESC-PTP <5%, the 2019 strategy classified 40% (7011/17,612) of patients into the deferral group. Table 1 shows the baseline characteristics by deferral and referral group based on the two strategies. Baseline characteristics significantly differed (*p* < 0.001) between the groups defined by the 2019 and 2024 strategies.

**Table 1.** **Baseline characteristics of patients based on the 2019 and 2024 strategies**.

Characteristics		Total (n = 17,612)	2019 strategy		*p*	2024 strategy		*p*
			Referral group	Deferral group		Referral group	Deferral group	
			n = 10,601	n = 7011		n = 9453	n = 8159	
Age^a^		58.27 ± 7.94	61.25 ± 8.15	53.76 ± 8.49	<0.001	60.73 ± 8.03	55.42 ± 8.63	<0.001
Male		9510 (54)	6679 (63)	2831 (40)	<0.001	5861 (62)	3649 (45)	<0.001
Diabetes		2149 (12)	1781 (17)	368 (5.2)	<0.001	1692 (18)	457 (5.6)	<0.001
Hypertension		8137 (46)	5216 (49)	2921 (42)	<0.001	4849 (51)	3828 (40)	<0.001
Hyperlipidemia		5125 (29)	3477 (33)	1648 (24)	<0.001	3280 (35)	1845 (23)	<0.001
Smoking		5970 (34)	3848 (36)	2122 (30)	<0.001	3611 (38)	2359 (29)	<0.001
Family history of CAD		3734 (21)	2555 (24)	1179 (17)	<0.001	2524 (27)	1210 (15)	<0.001
Symptom					<0.001			<0.001
	Nonanginal chest pain	7239 (41)	3350 (31)	3889 (56)		3290 (35)	3949 (48)	
	Atypical angina	6657 (38)	4325 (41)	2332 (33)		3705 (39)	2952 (36)	
	Typical angina	3716 (21)	2926 (28)	790 (11)		2458 (26)	1258 (16)	

CAD, coronary artery disease; 2019 strategy, risk assessment strategy recommended by 2019 European Society of Cardiology guidelines; 2024 strategy, risk assessment strategy recommended by 2024 European Society of Cardiology guidelines. Values are presented as n (%) unless stated otherwise. ^a ^years, mean ± standard deviation.

### 3.2 CAD on CCTA

The associations between CAD and risk groups identified in the 2019 and 2024 strategies are presented in Fig. 2. When defining obstructive CAD as ≥50% diameter stenosis (Fig. 2A), CCTA results showed obstructive, nonobstructive, and no CAD identified in 4086 (23%), 6583 (37%), and 6943 (40%) patients, respectively. Compared to patients in the deferral group, patients in the referral group had a higher prevalence of obstructive CAD and a lower prevalence of no CAD. Under both the 2019 and 2024 strategies, respectively, obstructive CAD was more common in the referral group (32% vs. 9.8% and 35% vs. 9.4%), while having no CAD was less common (29% vs. 55% and 26% vs. 56%) (*p* < 0.001 for all). As illustrated in Fig. 2B, similar trends were also observed when defining obstructive CAD based on ≥70% diameter stenosis. Compared to the 2019 strategy, the 2024 approach delivered a significantly higher diagnostic yield across all stenosis thresholds (>0%, ≥50%, and ≥70%), showing respective increases of 74% versus 71%, 35% versus 32%, and 19% versus 17% (*p* < 0.001 for all).

**Fig. 2.**
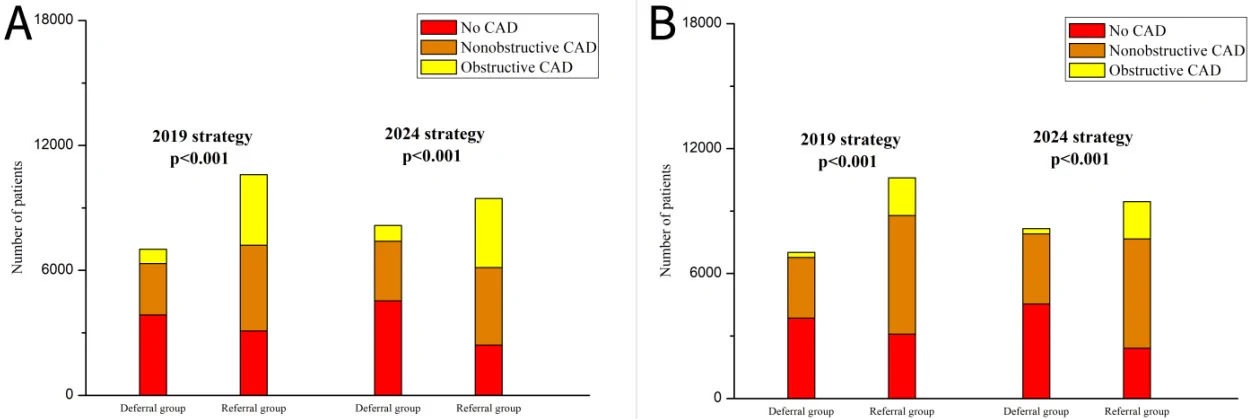
**Distribution of CAD in the deferral and referral groups determined by the 2019 and 2024 strategies**. Obstructive CAD was defined based on ≥50% (A) or ≥70% (B) diameter stenosis. CAD, coronary artery disease; 2019 strategy, risk assessment strategy recommended by 2019 European Society of Cardiology guidelines; 2024 strategy, risk assessment strategy recommended by 2024 European Society of Cardiology guidelines.

### 3.3 Follow-up and Subsequent Clinical Management

Patients were followed up for a median of 69 (interquartile range: 62 to 77) months, and 1922 (11%) patients were lost to follow-up. As shown in Table 2, 9.0% (1592/17,612) of patients experienced MACE, which consisted of a 3.9% mortality rate and nonfatal MI in 5.5% of patients. Fig. 3 shows the Kaplan–Meier estimates for MACE-free survival. Referral groups under both the 2019 and 2024 strategies faced a significantly elevated risk of MACE (log-rank *p* < 0.001), with HRs of 3.28 (95% CI: 2.98–3.63) and 3.64 (95% CI: 3.29–4.03), respectively. Similar results were also found when only considering death and nonfatal MI, respectively, as well as cardiovascular death and late revascularization (Table 2). The HRs for these endpoints were not significantly (*p* > 0.05) different between the 2019 and 2024 strategies, with the exception of late revascularization (Table 2). As baseline characteristics were similar between patients who were and were not lost to follow-up (Table 3), we assumed loss to follow-up was completely random. A sensitivity analysis excluding these patients yielded HRs of 3.50 (95% CI: 3.04–3.98) and 3.83 (95% CI: 3.41–4.29).

**Table 2.** **Association between study endpoints and groups according to the 2019 and 2024 strategies**.

		Total (n = 17,612)	2019 strategy			2024 strategy			*p* for comparison between two HRs
			Deferral group (n = 7011)	Referral group (n = 10,601)	HR^b^ (95% CI)	Deferral group (n = 8159)	Referral group (n = 9453)	HR^b^ (95% CI)	
Primary endpoint^a^		1592 (9.0)	274 (3.9)	1318 (12)	3.28 (2.98 to 3.63)	316 (3.9)	1276 (13)	3.64 (3.29 to 4.03)	0.149
Secondary endpoints									
	All-cause death	683 (3.9)	136 (1.9)	547 (5.2)	2.70 (2.29 to 3.16)	154 (1.9)	529 (5.6)	3.02 (2.59 to 3.47)	0.313
	Nonfatal MI	972 (5.5)	149 (2.1)	823 (7.8)	3.83 (3.41 to 4.27)	169 (2.1)	803 (8.5)	4.25 (3.71 to 4.83)	0.240
	Cardiovascular death	325 (1.8)	58 (0.8)	267 (2.5)	3.11 (2.45 to 3.85)	61 (0.7)	264 (2.8)	3.84 (3.04 to 4.81)	0.202
	Late revascularization	2074 (12)	392 (5.6)	1682 (16)	3.07 (2.91 to 3.24)	401 (4.9)	1673 (18)	3.81 (3.52 to 4.12)	<0.001

Values are presented as n (%) unless stated otherwise. MI, Myocardial infarction; HR, hazard ratio; CI, confidence interval; 2019 strategy, risk assessment strategy recommended by 2019 European Society of Cardiology guidelines; 2024 strategy, risk assessment strategy recommended by 2024 European Society of Cardiology guidelines. ^a^ Primary endpoint is defined as the composite of death and nonfatal MI. ^b^ Cox proportional hazards regression model is used to estimate HR comparing referral to deferral group.

**Fig. 3.**
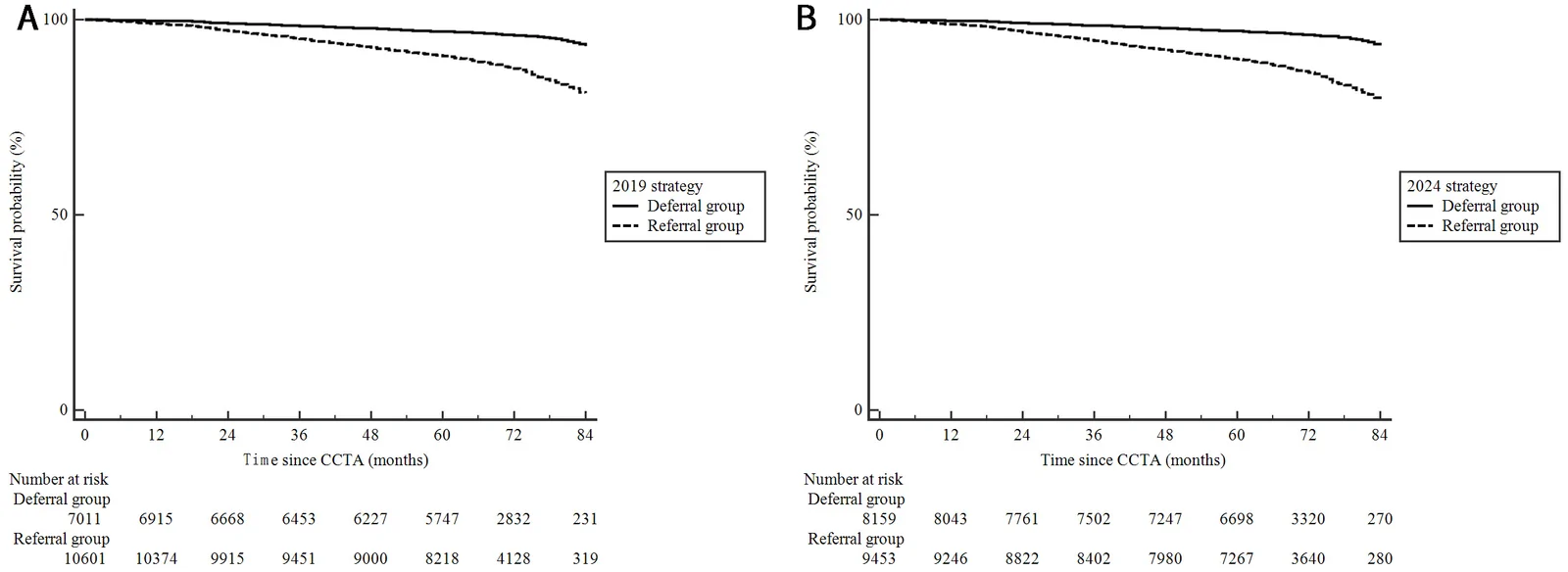
**Kaplan–Meier curves for major adverse cardiovascular event-free survival**. (A) 2019 strategy, log-rank *p* < 0.001. (B) 2024 strategy, log-rank *p* < 0.001. CCTA, coronary computed tomography angiography; 2019 strategy, risk assessment strategy recommended by 2019 European Society of Cardiology guidelines; 2024 strategy, risk assessment strategy recommended by 2024 European Society of Cardiology guidelines.

**Table 3.** **Baseline characteristics of patients based on loss to follow-up**.

Characteristics		Total (n = 17,612)	Loss to follow-up		*p*
			Yes	No	
			n = 1922	n = 15,690	
Age^a^		58.27 ± 7.94	58.43 ± 8.39	58.25 ± 7.98	0.353
Male		9510 (54)	1055 (55)	8455 (54)	0.419
Diabetes		2149 (12)	252 (13)	1897 (12)	0.210
Hypertension		8137 (46)	919 (48)	7218 (46)	0.139
Hyperlipidemia		5125 (29)	582 (30)	4543 (29)	0.237
Smoking		5970 (34)	669 (35)	5301 (34)	0.386
Family history of CAD		3734 (21)	436 (23)	3298 (21)	0.098
Symptom					0.117
	Nonanginal chest pain	7239 (41)	805 (42)	6434 (41)	
	Atypical angina	6657 (38)	746 (39)	5911 (38)	
	Typical angina	3716 (21)	371 (19)	3345 (21)	

CAD, coronary artery disease; 2019 strategy, risk assessment strategy recommended by 2019 European Society of Cardiology guidelines; 2024 strategy, risk assessment strategy recommended by 2024 European Society of Cardiology guidelines. Values are presented as n (%) unless stated otherwise. ^a ^years, mean ± standard deviation.

The associations between secondary endpoints and groups according to the 2019 and 2024 strategies are shown in Fig. 4. Compared with patients in the deferral group, patients in the referral group had higher rates of medication increases (2019 strategy: 30% vs. 5.3%; 2024 strategy: 33% vs. 4.9%), ICA (2019 strategy: 28% vs. 4.0%; 2024 strategy: 31% vs. 4.0%), and revascularization procedures (2019 strategy: 8.4% vs. 0.9%; 2024 strategy: 9.3% vs. 0.9%) (*p* < 0.001 for all).

**Fig. 4.**
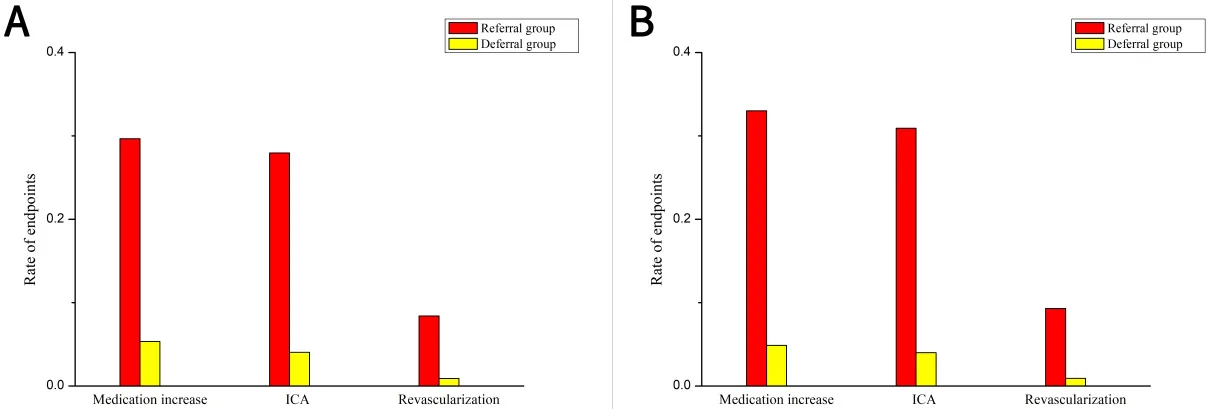
**Rates of secondary endpoints in the deferral and referral groups determined by the 2019 and 2024 strategies**. (A) 2019 strategy. (B) 2024 strategy. ICA, invasive coronary angiography; 2019 strategy, risk assessment strategy recommended by 2019 European Society of Cardiology guidelines; 2024 strategy, risk assessment strategy recommended by 2024 European Society of Cardiology guidelines.

### 3.4 Comparison of the Two Strategies by NRI

Tables 4,5,6 are the reclassification tables comparing the 2024 and 2019 strategies based on different definitions of positive patients. Based on ≥50% diameter stenosis (Table 4), the 2024 strategy correctly reclassified 1110 patients from the referral to the deferral group compared to the 2019 strategy. However, it incorrectly reclassified 37 of 13,526 negative patients from the deferral to the referral group. Of the 4086 positive patients, 2 were correctly assigned to the referral group, but 77 were incorrectly assigned to the deferral group. Thus, when comparing the 2024 and 2019 strategies for the RF-CL model, the NRI was 6.11% in all (*p* < 0.001), consisting of a negative NRI of 7.94 and a positive NRI of 1.83. Similar results were found for patients with a diameter stenosis of 70% (Table 5, NRI = 6.66%; *p* < 0.001) and those with 0% stenosis (Table 6, NRI = 5.29%; *p* < 0.001).

**Table 4.** **Reclassification table comparing the 2019 and 2024 strategies**.

		2024 strategy		Total	Reclassification^a^		NRI^b^	*p*
		Deferral group	Referral group		Up	Down		
2019 strategy								
Negative patients					0.27%	8.21%	6.11%	<0.001
	Deferral group	6285	37	6322				
	Referral group	1110	6094	7204				
Total		7395	6131	13,526				
Positive patients^c^					0.05%	1.88%		
	Deferral group	687	2	689				
	Referral group	77	3320	3397				
Total		764	3322	4086				

2019 strategy, risk assessment strategy recommended by 2019 European Society of Cardiology guidelines; 2024 strategy, risk assessment strategy recommended by 2024 European Society of Cardiology guidelines. ^a^ The classification of patients by the 2024 strategy was compared to that by the 2019 strategy. ^b^ NRI = [P(Up | Positive) – P(Down | Positive)] – [ P(Up | Negative) – P(Down | Negative)]. ^c ^A positive patient was defined as a patient had at least one lesion with ≥50% diameter stenosis.

**Table 5.** **Reclassification table comparing the 2019 and 2024 strategies**.

		2024 strategy		Total	Reclassification^a^		NRI^b^	*p*
		Deferral group	Referral group		Up	Down		
2019 strategy								
Negative patients					0.85%	8.15%	6.66%	<0.001
	Deferral group	6637	133	6770				
	Referral group	1268	7524	8792				
Total		7905	7657	15,562				
Positive patients^c^					0.63%	1.27%		
	Deferral group	228	13	241				
	Referral group	26	1783	1809				
Total		254	1796	2050				

2019 strategy, risk assessment strategy recommended by 2019 European Society of Cardiology guidelines; 2024 strategy, risk assessment strategy recommended by 2024 European Society of Cardiology guidelines. ^a^ The classification of patients by the 2024 strategy was compared to that by the 2019 strategy. ^b^ NRI = [P(Up | Positive) – P(Down | Positive)] – [ P(Up | Negative) – P(Down | Negative)]. ^c^ A positive patient was defined as a patient had at least one lesion with ≥70% diameter stenosis.

**Table 6.** **Reclassification table comparing the 2019 and 2024 strategies**.

		2024 strategy		Total	Reclassification^a^		NRI^b^	*p*
		Deferral group	Referral group		Up	Down		
2019 strategy								
Negative patients					0.43%	10.15%	5.29%	<0.001
	Deferral group	3827	30	3857				
	Referral group	705	2381	3086				
Total		4532	2411	6943				
Positive patients^c^					0.04%	4.47%		
	Deferral group	3150	4	3154				
	Referral group	477	7038	7515				
Total		3627	7042	10,669				

2019 strategy, risk assessment strategy recommended by 2019 European Society of Cardiology guidelines; 2024 strategy, risk assessment strategy recommended by 2024 European Society of Cardiology guidelines. ^a ^The classification of patients by the 2024 strategy was compared to that by the 2019 strategy. ^b^ NRI = [P(Up | Positive) – P(Down | Positive)] – [ P(Up | Negative) – P(Down | Negative)]. ^c^ A positive patient was defined as a patient had at least one lesion with >0% diameter stenosis.

Based on Fig. 2A and Table 4, we evaluated how each strategy defers CITs. Among the 17,612 patients evaluated by the 2019 strategy, 6322 were correctly assigned to the deferral group, and 689 were incorrectly assigned. Therefore, the 2019 strategy achieved a correct deferral rate of ([6322 – 689] / 17,612), which equates to 32.0%. This performance improved with the 2024 strategy, which resulted in a deferral rate of 38% ([7395 – 764] / 17,612). Based on data from Table 4, to avoid unnecessary CIT, the NNT comparing the 2024 to 2019 strategy was 17,612 / (1110 – 37), which equals ~17. According to the information in Fig. 1, adopting the 2024 instead of the 2019 strategy required collecting one additional set of risk factors for every 1.7 patients. Taking these factors into account, updating the 2019 strategy to the 2024 version prevented an unnecessary CIT, requiring only about 9 (17/2) additional risk factor collections.

Of the 4283 patients with an RF-CL ≤15% and an ESC-PTP between 5% and 15%, lowering the RF-CL to ≤5% reclassified 2075 patients to the referral group. Within this reclassified subgroup, only 14% (294/2075) had obstructive CAD (≥50% stenosis) and 4.4% (91/2075) experienced MACE.

## 4. Discussion

In this large-scale, CCTA-based cohort of Chinese patients with SCP suggestive of CCS, we demonstrated that under two current ESC guideline-recommended risk-stratification strategies, the deferral groups consistently showed lower rates of obstructive CAD, fewer clinical events, and less escalation of downstream management than their corresponding referral groups. Compared with the 2019 strategy, which applied the RF-CL model solely to patients with borderline ESC-PTP, the 2024 strategy applied it universally. This broader application proved more effective at safely deferring unnecessary CIT in low-risk patients. The superiority of the 2024 strategy may be primarily explained by its ability to identify a larger deferral group while maintaining a low prevalence of CAD, improving clinical care, and ensuring good patient outcomes.

SCP is a common manifestation of CCS; however, its underlying etiology spans a broad spectrum, from minimal myocardial damage to severe coronary anatomy, or even non-cardiac issues, with distinctly different management strategies and unique prognostic implications [1,2,7,25]. While CITs play a critical role in the management of patients with SCP suggestive of CCS, their increasing use—often with low diagnostic yields—strains healthcare systems. This highlights a critical need to better identify patients who can safely forgo further CIT [13]. To maximize the yield from limited resources, the ESC-PTP and RF-CL models have been developed [8,9], externally validated [10,11,12,13,14,15], and recommended by recent international guidelines [1,2,7].

While the ESC-PTP model has demonstrated very good calibration, with only minor overestimation of the prevalence of obstructive CAD [26], several comparative studies have shown that the RF-CL model offers better discrimination, calibration, and reclassification [10,12,13,14]. In line with these studies, the present study found that both the 2019 and 2024 strategies successfully identified patient groups with a low prevalence of CAD, clinical events, and downstream changes. However, the 2024 strategy, which applied the RF-CL model to all patients, safely and correctly deferred more CITs than the 2019 strategy, which only applied the RF-CL model to patients with borderline ESC-PTP. Additionally, the 2024 strategy demonstrated stronger links between patient groups and clinical outcomes, required more intensive follow-up care, and yielded positive NRIs according to various definitions of positive patients.

Based on recommendations from updated guidelines, further CITs are needed for all patients in the referral group, rather than the deferral group [1,2]. If applied in real clinical practice, the 2024 strategy would likely yield a higher diagnostic rate than the 2019 strategy. Comprehensive appraisal and synthesis of the available evidence is important, as the RF-CL model (based on symptoms and risk factors) is readily accessible in daily clinical practice [9]. Thus, by avoiding nine unnecessary requests for CIT-related risk factor information, replacing the 2019 strategy with the 2024 approach proved more efficient [12]. Collecting information on risk factors is a critical component of CCS management and is highly recommended by both the 2019 and 2024 guidelines; nonetheless, its integration into routine clinical practice can be hindered by variability and practical challenges.

Taken together, the findings of this study support the adoption of the 2024 strategy as a safe and effective approach for identifying patients with SCP suggestive of CCS who are unlikely to derive meaningful benefit from further CIT.

## 5. Strengths and Limitations

One of the major strengths of this study was its analysis of a large, long-term Chinese registry of CCTA data, enabling the evaluation of risk assessment strategies in real-world settings with substantial statistical power. In addition, our comprehensive analytic approach enabled us to evaluate the effectiveness and safety of risk assessment strategies through multiple parameters and endpoints. However, the findings of this study must be interpreted within the context of its design and accompanying limitations. First, this was an observational study. The clinical management of patients before and after CCTA was not protocol-driven and relied on local physicians, which could systematically inflate the association between referral group and events. Thus, whether the 2024 strategy translates into more appropriate downstream management decisions and better clinical outcomes remains to be determined in future studies, preferably randomized controlled trials (RCTs). Second, a formal cost effectiveness analysis (e.g., dollars per correctly deferred patient or per MACE avoided) is needed to justify the 2024 strategy of preventing unnecessary CIT in real-world resource-constrained settings. Third, the data were limited to a single Chinese registry, which restricts the generalizability of the results to other populations in different healthcare environments. Consequently, external validation in multi-ethnic cohorts is required prior to adopting these findings into clinical guidelines. Fourth, while recent guidelines [1,2] endorse the coronary artery calcium score (CACS)-weighted CL model for refining cardiac risk estimates [9], this approach has notable drawbacks. Incorporating CACS requires an extra imaging test, which entails additional radiation exposure, increased healthcare costs, and diagnostic delays. These factors ultimately contradict the primary goal of safely identifying low-risk patients who can forgo further CIT [17]. Finally, the present analysis focused on CAD detected by CCTA. Because CCTA has a well-documented high negative predictive value, it reliably rules out obstructive CAD as well as invasive coronary angiography [17]. Consistent results were achieved across various diameter stenosis thresholds (e.g., ≥50%, ≥70%, and >0%) used to define positive cases.

## 6. Conclusions

For patients with SCP suspected of CCS, both the 2019 and 2024 strategies identified patient groups with a lower risk of CAD, MACE, and the need for intensive clinical management. The 2024 strategy shows greater promise for avoiding unnecessary CIT safely compared to the 2019 approach. However, future RCTs are required to evaluate the cost-effectiveness of these strategies for deferring CIT in patients presenting with SCP related to obstructive CAD.

## Data Availability

The datasets used and analyzed during the current study are available from the corresponding author on reasonable request.

## References

[1] Gulati M, Levy PD, Mukherjee D, Amsterdam E, Bhatt DL, Birtcher KK (2021). 2021 AHA/ACC/ASE/CHEST/SAEM/SCCT/SCMR Guideline for the Evaluation and Diagnosis of Chest Pain: A Report of the American College of Cardiology/American Heart Association Joint Committee on Clinical Practice Guidelines. Journal of the American College of Cardiology.

[2] Vrints C, Andreotti F, Koskinas KC, Rossello X, Adamo M, Ainslie J (2024). 2024 ESC Guidelines for the management of chronic coronary syndromes: Developed by the task force for the management of chronic coronary syndromes of the European Society of Cardiology (ESC) Endorsed by the European Association for Cardio-Thoracic Surgery (EACTS). European Heart Journal.

[3] Weir-McCall JR, Williams MC, Shah ASV, Roditi G, Rudd JHF, Newby DE (2023). National Trends in Coronary Artery Disease Imaging: Associations With Health Care Outcomes and Costs. JACC. Cardiovascular Imaging.

[4] Bularga A, Saraste A, Fontes-Carvalho R, Holte E, Cameli M, Michalski B (2021). EACVI survey on investigations and imaging modalities in chronic coronary syndromes. European Heart Journal. Cardiovascular Imaging.

[5] Wong M, Dai Y, Ge J (2024). Pan-vascular disease: what we have done in the past and what we can do in the future?. Cardiology Plus.

[6] Trimarchi G, Carerj ML, Zito C, Bella GD, Taverna G, Cusmà Piccione M (2025). Epicardial Adipose Tissue: A Multimodal Imaging Diagnostic Perspective. Medicina.

[7] Knuuti J, Wijns W, Saraste A, Capodanno D, Barbato E, Funck-Brentano C (2020). 2019 ESC Guidelines for the diagnosis and management of chronic coronary syndromes. European Heart Journal.

[8] Knuuti J, Ballo H, Juarez-Orozco LE, Saraste A, Kolh P, Rutjes AWS (2018). The performance of non-invasive tests to rule-in and rule-out significant coronary artery stenosis in patients with stable angina: a meta-analysis focused on post-test disease probability. European Heart Journal.

[9] Winther S, Schmidt SE, Mayrhofer T, Bøtker HE, Hoffmann U, Douglas PS (2020). Incorporating Coronary Calcification Into Pre-Test Assessment of the Likelihood of Coronary Artery Disease. Journal of the American College of Cardiology.

[10] Rasmussen LD, Williams MC, Newby DE, Dahl JN, Schmidt SE, Bøttcher M (2023). External validation of novel clinical likelihood models to predict obstructive coronary artery disease and prognosis. Open Heart.

[11] Baskaran L, Yan L, Tan CS, Ho WW, Tan SY, Williams MC (2024). Evaluating the American Heart Association/American College of Cardiology Guideline-Recommended and Contemporary Pretest Probability Models in a Mixed Asian Cohort: The Contribution of Coronary Artery Calcium. Journal of the American Heart Association.

[12] Brix GS, Rasmussen LD, Rohde PD, Schmidt SE, Nyegaard M, Douglas PS (2024). Calcium Scoring Improves Clinical Management in Patients With Low Clinical Likelihood of Coronary Artery Disease. JACC. Cardiovascular Imaging.

[13] Winther S, Schmidt SE, Foldyna B, Mayrhofer T, Rasmussen LD, Dahl JN (2022). Coronary Calcium Scoring Improves Risk Prediction in Patients With Suspected Obstructive Coronary Artery Disease. Journal of the American College of Cardiology.

[14] Zhou J, Li C, Cong H, Duan L, Wang H, Wang C (2022). Comparison of Different Investigation Strategies to Defer Cardiac Testing in Patients With Stable Chest Pain. JACC. Cardiovascular Imaging.

[15] Zhou J, Zhao J, Li Z, Cong H, Wang C, Zhang H (2022). Coronary calcification improves the estimation for clinical likelihood of obstructive coronary artery disease and avoids unnecessary testing in patients with borderline pretest probability. European Journal of Preventive Cardiology.

[16] Zhou J, Li C, Zhang H, Liu C, Yang J, Zhao J (2023). Association between Coronary Artery Disease Reporting and Data System-recommended Post-Coronary CT Angiography Management and Clinical Outcomes in Patients with Stable Chest Pain from a Chinese Registry. Radiology.

[17] Cury RC, Leipsic J, Abbara S, Achenbach S, Berman D, Bittencourt M (2022). CAD-RADS™ 2.0 - 2022 Coronary Artery Disease-Reporting and Data System: An Expert Consensus Document of the Society of Cardiovascular Computed Tomography (SCCT), the American College of Cardiology (ACC), the American College of Radiology (ACR), and the North America Society of Cardiovascular Imaging (NASCI). Journal of Cardiovascular Computed Tomography.

[18] Thygesen K, Alpert JS, Jaffe AS, Chaitman BR, Bax JJ, Morrow DA (2018). Fourth Universal Definition of Myocardial Infarction (2018). Journal of the American College of Cardiology.

[19] Hicks KA, Mahaffey KW, Mehran R, Nissen SE, Wiviott SD, Dunn B (2018). 2017 Cardiovascular and Stroke Endpoint Definitions for Clinical Trials. Journal of the American College of Cardiology.

[20] Boden WE, Kaski JC, Al-Lamee R, Weintraub WS (2022). What constitutes an appropriate empirical trial of antianginal therapy in patients with stable angina before referral for revascularisation?. Lancet.

[21] Simader FA, Rajkumar CA, Foley MJ, Ahmed-Jushuf F, Chotai S, Bual N (2024). Symptoms as a Predictor of the Placebo-Controlled Efficacy of PCI in Stable Coronary Artery Disease. Journal of the American College of Cardiology.

[22] Al-Lamee R, Thompson D, Dehbi HM, Sen S, Tang K, Davies J (2018). Percutaneous coronary intervention in stable angina (ORBITA): a double-blind, randomised controlled trial. Lancet.

[23] Altman DG, Bland JM (2003). Interaction revisited: the difference between two estimates. BMJ (Clinical Research Ed.).

[24] Alba AC, Agoritsas T, Walsh M, Hanna S, Iorio A, Devereaux PJ (2017). Discrimination and Calibration of Clinical Prediction Models: Users' Guides to the Medical Literature. JAMA.

[25] Winchester DE, Maron DJ, Blankstein R, Chang IC, Kirtane AJ, Kwong RY (2023). ACC/AHA/ASE/ASNC/ASPC/HFSA/HRS/SCAI/SCCT/SCMR/STS 2023 Multimodality Appropriate Use Criteria for the Detection and Risk Assessment of Chronic Coronary Disease. Journal of the American College of Cardiology.

[26] Winther S, Schmidt SE, Rasmussen LD, Juárez Orozco LE, Steffensen FH, Bøtker HE (2021). Validation of the European Society of Cardiology pre-test probability model for obstructive coronary artery disease. European Heart Journal.

